# A Randomized Controlled Trial to Examine the Relationship Between Peer Mentoring for Physical Activity and Cardiometabolic Health

**DOI:** 10.5334/gh.1268

**Published:** 2023-10-05

**Authors:** Moradeke Bamgboye, David Adeyemi, Emmanuel Agaba, Susan Yilme, Clement A. Adebamowo, Sally N. Adebamowo

**Affiliations:** 1 Department of Physiology, University of Maryland School of Medicine, Baltimore, Maryland, USA; 2 Clinton Health Access Initiative, Abuja, NG; 3 Univeristy of Jos, Jos, NG; 4 Institute of Human Virology Nigeria, Abuja, NG; 5 Department of Epidemiology and Public Health, University of Maryland School of Medicine, Baltimore, Maryland, USA; 6 University of Maryland Comprehensive Cancer Center, University of Maryland School of Medicine, Baltimore, Maryland, USA

**Keywords:** peer mentoring, physical activity, cardiometabolic health, randomized controlled trial

## Abstract

**Background:**

Studies examining the effect of peer mentoring on physical activity levels have been conducted in mostly elderly and young populations, and the results have been inconsistent. This study examined the impact of one-on-one peer mentoring on physical activity and cardiometabolic parameters in urban adults.

**Methods:**

The study participants were 353 overweight or obese adults residing in Abuja, Nigeria. They were randomized into two groups, peer-mentored (n = 128) and a control (n = 225) group. All the participants received nutritional counseling and were invited to attend instructor-led physical activity sessions each week for six months. Differences in the frequency, duration, and intensity of physical activity and cardiometabolic parameters, including body fat, muscle mass and lipids, were evaluated within and between the groups with *t* and rank sum tests.

**Results:**

At the end of the study period, the average time spent on physical activity increased significantly by 14% (*p* = 0.006), and the average time spent on vigorous physical activity increased by 99% (*p* = 0.003) compared to baseline for participants in the peer-mentored group. For those in the control group, the average time spent on physical activity decreased significantly by 7% (*p* = 0.03), while the average time spent on vigorous physical activity increased by 30%, but this was not statistically significant (*p* = 0.34). In both groups, there were significant improvements in the total cholesterol, low- and high-density lipoproteins and triglycerides levels, at the end of the study period, compared to baseline.

**Conclusions:**

In these overweight or obese adults, we observed that peer mentoring was positively associated with increased physical activity. Peer mentoring also holds great promise for improving cardiometabolic health and promoting a healthy lifestyle in adults.

## Introduction

Physical activity is an important modifiable determinant of health. The World Health Organization’s (WHO) physical activity recommendation for adults aged 18–64 years is at least 150–300 minutes of moderate-intensity aerobic physical activity or at least 75–150 minutes of vigorous-intensity aerobic physical activity or an equivalent combination of moderate- and vigorous-intensity activity per week [1]. Studies have shown that adults who meet the recommended physical activity levels have reduced risks of cardiometabolic disorders, cancer, and all-cause mortality [2,3,4,5,6,7,8,9,10,11]. In spite of the well-recognized health benefits of physical activity, global estimates indicate that one in four adults does not meet the WHO recommended physical activity levels [1]. In sub-Saharan Africa (SSA), there is evidence that increasing urbanization and westernization are associated with reduced levels of physical activity [12,13]. A previous study conducted in Nigeria, the most populous country in SSA, showed that over 80% of urban adults did not meet the WHO recommendation for physical activity [14]. Given the established health benefits associated with physical activity, it is imperative to incorporate strategies to increase physical activity levels, especially in SSA, where the prevalence of cardiometabolic disorders is increasing [15,16].

Peer mentoring is a relatively inexpensive and adaptable strategy to increase physical activity levels [17]. Peer mentoring for physical activity utilizes the motivational impact of peer-to-peer social interaction and support to increase individual and group participation in physical activity. The effect of peer mentoring on physical activity has mostly been studied in adolescents in school-based settings [18,19,20] or older adults [21,22,23] in high-income countries, and the results have been conflicting. There are no studies on the effects of one-on-one peer mentoring on physical activity among professional adults. This study was conducted to determine the effects of one-on-one peer mentoring on the frequency, duration, and intensity of physical activity and cardiometabolic parameters among overweight and obese urban professional adults in Abuja, Nigeria.

## Methods

### Study Design

This was a randomized controlled trial conducted to evaluate the hypothesis that one-on-one peer mentoring was associated with increased frequency, duration, and intensity of physical activity and cardiometabolic parameters among overweight and obese adults. The study evaluated the effects of peer mentoring on physical activity levels and cardiometabolic parameters, including anthropometric characteristics (weight, body-mass index [BMI], waist and hip circumferences), body fat (total body fat, visceral fat, skeletal muscle), and blood lipid profiles. Participants were randomized using a 1:2 ratio into two groups, the peer-mentor group and the control group. Participants assigned to the peer-mentor group were allowed to identify an individual from their group to be paired with. At baseline, participants in both groups received nutritional counseling based on the healthy eating pyramid [24] and were invited to attend two hours of moderate-to-vigorous physical activity led by a trained physical activity instructor at a fitness center per week (individually for the control group and in pairs for peer-mentor group participants) for six months. Participants were also encouraged to increase, record, and report unsupervised leisure-time physical activity (at home and at work).

### Study Population

Participants were recruited from the federal secretariat in Abuja, central Nigeria, from July 2013 to May 2014. A random enumerated list of offices at the federal secretariat was generated; at each office assigned an odd number, workers and adult visitors were approached to be part of the study cohort. Informed consent was obtained from interested participants who were 18 to 64 years old, overweight or obese, had no contraindications to exercise, were not already on any type of weight loss intervention, were not pregnant or had not planned to become pregnant, travel, change jobs, or residence during the study period.

### Ethics Statement

The study was conducted according to the Nigerian National Code for Health Research Ethics. Ethical approval to conduct this study was obtained from the Institute of Human Virology, Nigeria, Health Research Ethics Committee and the National Health Research Ethics Committee (NHREC) of Nigeria. Written informed consent was obtained from all participants before enrollment in the study.

### Sociodemographic Characteristics

At baseline, data was collected on ethnicity, religion, marital status, type of profession, and level of education. Socioeconomic status was determined using the Filmer-Pritchett wealth index [25], by constructing a linear index from asset ownership indicators, such as vehicles, information on household type, and the number of residents, using principal component analysis to derive weights.

### Anthropometric and Body Composition Measurements

Trained research associates measured participant anthropometry at baseline and six months later. Participants’ height and weight were measured with a rigid tape measure. Height was measured without shoes. Weight, total body fat, and visceral and skeletal muscle mass were measured using the Omron HBF-510 W Full Body Sensor Body Composition Monitor Scale after the removal of all heavy outer garments and the emptying of pockets. Waist circumference was measured at the widest point between the bottom of the rib cage, and the iliac crest and hip circumference was measured at the widest point of the hip. BMI was calculated as the ratio of weight (kg) to height (m^2^) and categorized using WHO cut points in units of kg/m^2^ (normal weight = 18.5–24.9, overweight = 25.0–29.9 and obese ≥ 30) [26].

### Physical activity levels

Trained research associates monitored the frequency, duration, and intensity of the participants’ physical activity at the fitness center. Interviewer-administered questionnaires were used to collect data on the participants leisure-time physical activity at baseline and six months later. Physical activity was recorded as the amount of time spent per week on each of the following activities: running, jogging, riding a bicycle, dancing, table tennis, soccer, basketball, squash, golfing, hiking, swimming, other aerobic exercise, weightlifting, and walking to and from work. MET minutes (METmin/wk) and hours (METhr/wk) per week were calculated by assigning MET values to each activity based on the compendium of physical activity intensities [27] and multiplying them by the duration and frequency of leisure time activity per week. Moderate and vigorous physical activities were categorized as activities with 3.5–6.0 METS and >6.0 METS, respectively. Physical activity levels were also categorized as low (< 200 METmins/wk), moderate (200–450 METmins/wk) and sufficient (>450 METmins/wk), based on WHO definitions.

### Biochemical Markers

Blood samples were collected by a licensed phlebotomist at baseline and six months later; plasma lipoprotein levels were assessed and reported in mg/dl. Lipid profiles were classified according to the American College of Cardiology/American Heart Association clinical guidelines as normal (total cholesterol < 200 mg/dl, triglycerides < 150 mg/dl, low-density lipoproteins (LDL) < 130 mg/dl and high-density lipoproteins (HDL) > 60 mg/dl) and dyslipidemia (cholesterol ≥ 200 mg/dl or triglycerides ≥ 150 mg/dl or LDL ≥ 130 mg/dl or HDL < 40 mg/dl) [28].

### Statistical Analysis

Sample size calculations were based on the ability to detect a 10% change in minutes spent on leisure-time physical activity and a 5% change in weight. A sample size of 62 participants per group was predicted to have a power of 80% to detect these changes at a 5% significance level. Accounting for a possible but unlikely 20% attrition rate brought the number to 75 per group. The data was assessed for normality to ensure that the assumptions that informed the analyses were met. *t*-tests were used to assess the difference between the two groups for data that met the criteria for normality. Otherwise, the Wilcoxon rank sum test was used to compare data between groups. Changes in METmins/wk, moderate and vigorous physical activity levels, cholesterol, triglycerides, LDL, high-density lipoproteins (HDL), and anthropometric measures were analyzed within groups with the paired *t*-test. Per-protocol analyses were planned, and all analyses were conducted with SAS statistical software.

## Results

**Figure 1:**
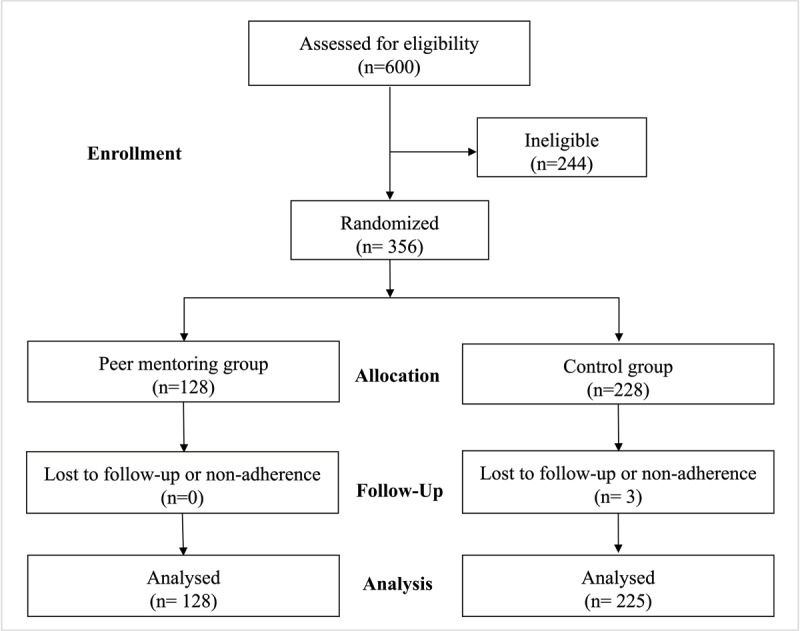
Participant flow chart.

The study flow chart is shown in Figure 1. Three hundred and fifty-three overweight or obese participants (peer-mentor group, n = 128; control group, n = 225) were included in the analysis. The sociodemographic characteristics of the study participants at baseline are shown in Table 1. Age, gender, marital status, education, occupation, smoking, and alcohol use were similar between the groups. Socioeconomic status differed between the groups.

**Table 1:** Socio-demographic Characteristics of the Study Participants at Baseline.


	TOTAL (N = 353)	PEER-MENTOR (N = 128)	CONTROL (N = 225)	*P*-VALUE

**Age, mean ± SD**	42.69 ± 8.39	42.99 ± 8.68	42.51 ± 8.23	0.60

**Age, %**				0.27

– 20–30 years	6.0	8.6	4.4	

– 30–40 years	31.2	26.6	33.8	

– 40–50 years	38.2	39.0	37.8	

– 50–60 years	24.6	25.8	24.0	

**Gender, %**				0.75

– Male	33.0	32.8	33.0	

– Female	67.0	67.2	67.0	

**Marital Status, %**				0.33

– Married	77.6	80.5	76.0	

– Not married	22.4	19.5	24.0	

**Highest level of education, %**				0.18

– Secondary school	5.4	7.8	4.0	

– Post-secondary school	94.6	92.2	96.0	

**Occupation, %**				0.47

– Professional	92.4	93.0	92.0	

– Others^*^	7.6	7.0	8.0	

**Socio-economic status, %**				<0.001

– Low	22.7	17.2	25.8	

– Middle	25.2	14.8	31.1	

– High	52.1	68.0	43.1	

**Smoking, %**				0.13

– Current smoker	2.8	0.8	4.0	

– Former smoker	7.1	5.5	8.0	

– Never smoked	90.1	93.7	88.0	

**Alcohol, %**				0.34

– Current drinker	54.1	60.0	52.2	

– Former drinker	8.7	4.0	10.2	

– Never drank	37.2	36.0	37.6	


Professional occupation includes accountants, lawyers, scientists, teachers; other* occupations include carpenters, homemakers, unemployed.

**Figure 2:**
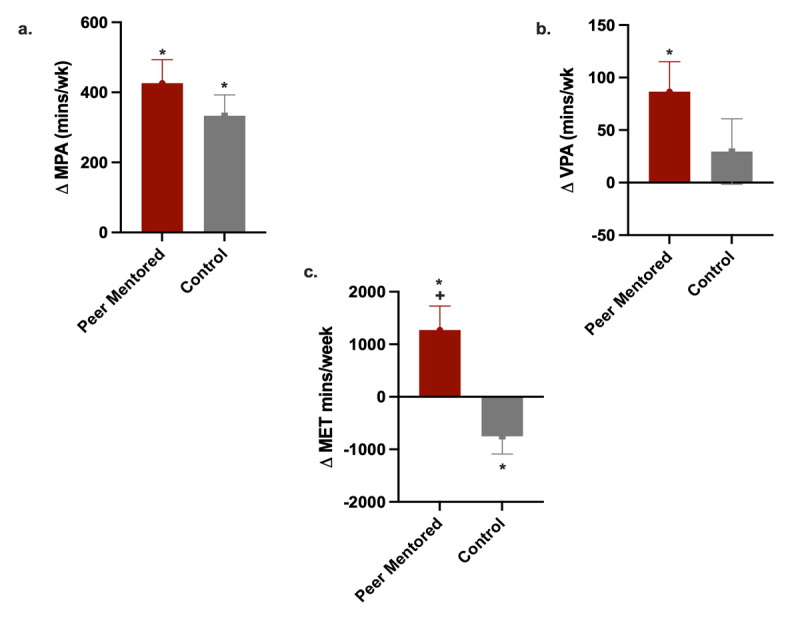
Change in physical activity by group. **a.** Change in moderate-intensity physical activity (MPA). **b.** Change in vigorous-intensity physical activity (VPA). **c.** Change in total MET minutes spent of physical activity per week.

The anthropometric and clinical characteristics of the study participants at baseline are shown in Table 2. The characteristics were similar between the groups. Table 3 shows the participants’ physical activity levels at baseline and follow-up visits (six months after baseline) and the results of tests comparing the mean values of physical activity duration within and between the groups. At baseline, the peer-mentor group reported lower physical activity levels compared to the control group. However, by the end of the six-month study period, the physical activity METS minutes/week increased by 14% (*p* = 0.006) for those in the peer-mentor group and decreased by 7% (*p* = 0.03) for those in the control group (Figure 2). The average minutes spent on moderate physical activity at baseline versus the end of the study period significantly increased by 124% (347 vs. 776, *p* =< 0.0001) in the peer-mentor group and 68% (480 vs. 804 p =< 0.001) in the control group. However, the average minutes spent on vigorous physical activity at baseline versus the end of the study period significantly increased by 99% (85 vs. 169, p = 0.03) in the peer-mentor group. The average minutes spent on vigorous physical activity at baseline versus the end of the study period also increased by 30% (120 vs. 155, p-value = 0.34) in the control group, but this was not statistically significant. The proportion of participants with sufficient physical activity levels after the study intervention was also significantly increased in both groups (Table 4).

**Table 2:** Anthropometric and Clinical Characteristics of the Study Participants at Baseline.


	TOTAL (N = 353)	PEER-MENTOR (N = 128)	CONTROL (N = 225)

	MEAN ± SD		

Weight, kg	88.89 ± 14.97	89.46 ± 14.21	88.56 ± 15.39

Body Mass Index, kg/m^2^	32.32 ± 5.97	33.15 ± 6.67	31.85 ± 5.49

Waist circumference	107.10 ± 11.49	108.46 ± 11.31	106.33 ± 11.54

Hip circumference	116.26 ± 10.33	117.02 ± 10.04	115.82 ± 10.49

Total body fat	40.73 ± 9.06	41.26 ± 8.35	40.43 ± 9.44

Visceral fat	11.37 ± 4.17	11.47 ± 4.01	11.31 ± 4.27

Skeletal muscle	26.35 ± 5.01	26.18 ± 4.73	26.45 ± 5.16

Total cholesterol	206.07 ± 43.68	204.06 ± 43.37	207.23 ± 43.92

Triglycerides	129.06 ± 71.38	123.68 ± 59.83	132.16 ± 77.22

Low density lipoprotein	135.66 ± 40.16	133.81 ± 41.00	136.77 ± 39.71

High density lipoprotein	44.30 ± 14.64	44.41 ± 9.90	44.23 ± 16.83

Systolic blood pressure	127.62 ± 20.56	128.18 ± 19.01	127.30 ± 21.42

Diastolic blood pressure	84.66 ± 13.64	85.52 ± 11.79	84.17 ± 14.59

	**%**		

Body Mass Index, kg/m^2^			

– Overweight	36.75	30.08	40.67

– Obese	63.25	69.92	59.33

Lipid Profile			

– Normal Lipid Profile	26.63	26.56	26.67

– Dyslipidemia	73.37	73.44	73.33

Cholesterol, mg/dl			

– Normal, < 200	45.82	48.03	44.55

– Borderline, 200 –< 240	34.87	36.22	34.09

– High, ≥ 240	19.31	15.75	21.36

Triglycerides, mg/dl			

– Normal, < 150	70.61	70.08	70.91

– Borderline, 150 –< 200	15.27	17.32	14.09

– High, ≥ 200	14.12	12.60	15.00

Low density lipoprotein (LDL), mg/dl			

– Normal, 100 –< 130	20.12	22.76	18.54

– Borderline, 130 –< 160	55.18	54.47	55.61

– High, ≥ 160	24.70	22.76	25.85

High density lipoprotein (HDL), mg/dl			

– Ideal, ≥ 60	10.40	7.87	11.87

– Moderate, ≥ 40–60	26.30	29.92	24.20

– Low, < 40	63.29	62.20	63.93

Medical history			

– Family history of hypertension	42.9	40.1	44.4

– Family history of diabetes	23.0	23.6	22.7

– Doctor diagnosed hypertension	26.6	26.6	26.7

– Doctor diagnosed diabetes	6.2	4.7	7.1


**Table 3:** Participants Baseline and Follow-up Physical Activity, by Group.


PHYSICAL ACTIVITY(MINUTES)	PEER MENTORED GROUP				CONTROL GROUP				*P*-VALUE^#^
	
	BASELINE	FOLLOW-UP	DIFFERENCE	*P*-VALUE*	BASELINE	FOLLOW-UP	DIFFERENCE	*P*-VALUE*	

Weekly METS	9443.8 ± 4123.9	10750.3 ± 3751.4	+ 21.7	0.006	11131.4 ± 4304.4	10366.3 ± 3052.9	–12.8	0.03	0.0005

Duration of moderate physical activity	347.1 ± 458.9	776.0 ± 697.7	+ 428.9	<0.0001	480.2 ± 577.8	804.6 ± 800.4	+ 324.4	<0.001	0.30

Duration of vigorous physical activity	85.0 ± 213.2	169.3 ± 332.9	+ 81.6	0.003	120.3 ± 368.4	155.6 ± 442.1	+ 35.3	0.34	0.18


Difference = difference between means. *P*-value* = P-value comparing the mean values within each group, with a paired t-test. *P*-value^#^ = P-value comparing the difference in mean values between the groups, with a t-test.

**Table 4:** Comparison of sufficient physical activity within groups, at baseline and 6 months.


SUFFICIENT ACTIVITY, %	PEER-MENTOR	*P*-VALUE	CONTROL	*P*-VALUE

– Baseline	67.2	<0.0001	75.1	<0.0001

– 6 months	93.8		90.7	


**Figure 3:**
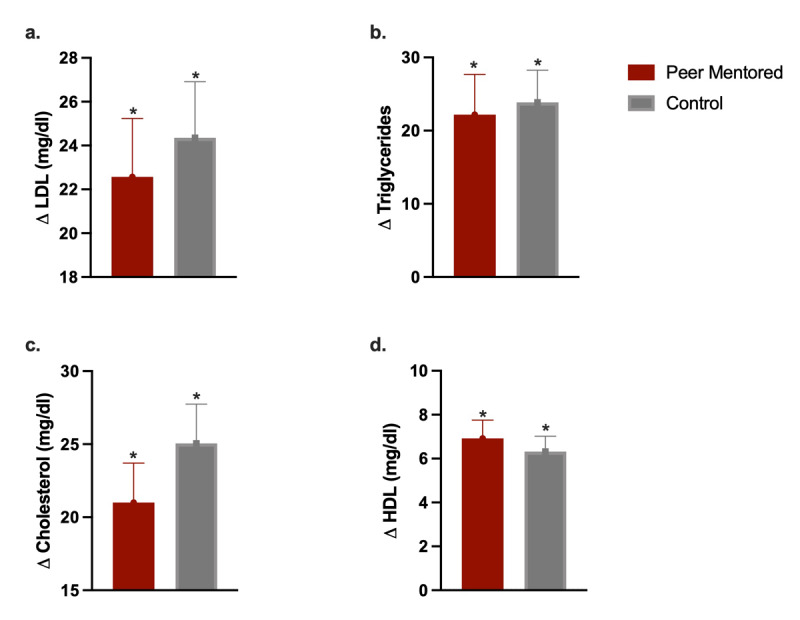
Change in cardiometabolic parameters (lipid profile) by group. **a.** Change in low-density lipoproteins (LDL). **b.** Change in triglycerides. **c.** Change in cholesterol. **d.** Change in high-density lipoproteins (HDL).

Both groups had significant reductions in waist and hip circumferences and nonsignificant reductions in body weight and visceral fat (Table 5). The levels of LDL, cholesterol, and triglycerides were significantly reduced, and the level of HDL was significantly increased in both groups at the end of the study period compared to baseline (Figure 3).

**Table 5:** Impact of physical activity on participants cardiometabolic parameters.


CARDIOMETABOLIC PARAMETERS	PEER MENTORED GROUP				CONTROL GROUP				*P*-VALUE^#^
	
	BASELINE	FOLLOW-UP	DIFFERENCE	*P*-VALUE	BASELINE	FOLLOW-UP	DIFFERENCE	*P*-VALUE	

Weight, kg	89.5 ± 14.2	89.1 ± 14.5	–0.4	0.24	88.6 ± 15.3	87.8 ± 14.7	–0.8	0.07	0.42

BMI, kg/m^2^	33.2 ± 6.6	33.0 ± 6.6	–0.2	0.23	31.9 ± 5.4	31.6 ± 5.3	–0.3	0.07	0.48

Waist-Hip Ratio	0.927 ± 0.05	0.910 ± 0.07	–0.017	0.002	0.919±0.06	0.915 ± 0.07	–0.0009	0.83	0.014

Total body fat, %	41.3 ± 8.3	41.4 ± 9.2	+ 0.1	0.63	40.4 ± 9.4	39.6 ± 9.7	–0.8	0.02	0.09

Visceral fat, %	11.5 ± 4.0	11.3 ± 3.6	–0.2	0.55	11.3 ± 4.2	11.1 ± 3.8	+ 0.2	0.93	0.67

Skeletal muscle, %	26.2 ± 4.7	26.3 ± 4.8	+ 0.1	0.84	26.5 ± 5.1	26.8 ± 5.3	+ 0.3	0.11	0.49

T-Chol, mg/dl	204.1 ± 43.3	183.8 ± 38.8	–20.3	<0.0001	207.2 ± 43.9	182.4 ± 40.4	–24.8	<0.0001	0.29

TG, mg/dl	123.7 ± 59.8	101.1 ± 45.0	–22.6	<0.0001	132.2 ± 77.2	102.4 ± 50.5	–29.8	<0.0001	0.81

LDL-Chol	133.8 ± 41.0	113.3 ± 36.1	–20.5	<0.0001	136.8 ± 39.7	113.0 ± 37.3	–23.8	<0.0001	0.63

HDL-Chol	44.4 ± 9.9	50.1 ± 7.6	+ 5.7	<0.0001	44.2 ± 16.8	48.9 ± 8.3	+ 4.7	<0.0001	0.72


Difference = difference between means. *P*-value* = P-value comparing the mean values within each group, with a paired t-test. *P*-value^#^ = P-value comparing the difference in mean values between the groups, with a t-test.

## Discussion

In this study, we observed that one-on-one peer mentoring was positively associated with increased duration of physical activity in overweight and obese adults. Health education and access to instructor-led physical activity sessions were associated with an increase in total time spent on physical activity and moderate physical activity (MPA) for both groups. At the end of the study period, the average amount of time spent on vigorous physical activity (VPA) levels significantly increased by 99% for participants in the peer-mentored group compared to baseline.

Our results are similar to those in other studies [21,22,29,30,31]. In a study comparing 81 sedentary adults randomized to either peer-delivered support for physical activity or basic education, gym membership, or a pedometer for self-monitoring, both groups had similar improvements in MPA at four months, while the group supplemented with peer support had significantly more MPA at 18 months [21]. In another study with 11 peer mentors and 44 participants with developmental disabilities, it was shown that a seven-month program consisting of peer mentoring, health education, and supervised physical activity was associated with increased exercise frequency [29]. Castro et al. also showed that trained peer volunteers can effectively promote physical activity in adults over 50 years of age through telephone-based advice [22]. Other studies also showed peer mentoring was associated with increased physical activity in children [30,31].

However, some studies that compared the effectiveness of physical activity interventions delivered by peer mentors versus other mentors showed improvement in physical activity but no difference between the groups. In one study that examined changes in physical activity over four months among individuals with diabetes, there were no significant differences in outcomes between groups led by professionals versus peers [32]. Similarly, another study that examined the effectiveness of an eight-month fitness program among adults showed that mentoring by peers versus young students were both associated with improved physical fitness, but there were no significant differences in the fitness measures between the groups [23]. These findings suggest that mentoring is beneficial for increasing physical activity and fitness levels.

Only a few studies have evaluated changes in anthropometric or cardiometabolic parameters after physical activity interventions. In one study, a six-month physical activity program was associated with 2.6 pounds of weight loss (1.18 kg) among overweight and obese adults with developmental disabilities (n = 44) [29]. Another study found that a four-month physical activity intervention was associated with statistically significant changes in weight, waist circumference, blood pressure, and resting heart rate among diabetic adults. However, there were no significant differences in outcomes between the participants led by professionals, n = 157, and those led by peers, n = 63 [32]. In the present study, we found significant improvements in cardiometabolic parameters, including waist circumference, blood pressure, and plasma lipid profiles, in both groups. Our results on changes in plasma lipid profile with minimal changes in weight are similar to those in other studies, which showed an association between moderate-to-vigorous intensity physical activity and improved plasma lipid profiles, even in the absence of weight loss [33,34]. These improvements in clinically relevant health metrics further reinforce the benefits of physical activity interventions for cardiometabolic health.

Our study has some limitations. We observed that those in the peer-mentor group had higher socioeconomic status compared to those in the control group. This may be because the peers were self-selected, and participants probably chose people from their social class to be paired with. Though we randomized participants to either group to reduce bias, we cannot rule out selection bias in this study. Nonetheless, our findings contribute important knowledge to the field by examining an indigenous African population and employing one-on-one mentoring by self-selected peers. We used interviewer-administered questionnaires to collect data on the participants leisure-time physical activity at baseline and six months, which may have led to an overestimation of their physical activity levels. However, since this procedure was the same for all participants, it could not lead to random errors and variability between the groups. Moreover, we observed and recorded moderate and vigorous physical activity levels during instructor-led sessions at a fitness center. Although we did not evaluate the cost-effectiveness of the intervention, this mentoring structure is inexpensive because it does not require training individuals as mentors to engage with the study participants, and it mimics real-life dynamics where adults are more likely to engage in physical activity with peers of their choice.

In conclusion, our study shows that peer mentoring was positively associated with increased physical activity levels among overweight and obese adults. Peer mentoring also holds great promise for improving cardiometabolic health and promoting a healthy lifestyle in adults.

## References

[1] World Health Organization. WHO guidelines on physical activity and sedentary behaviour. World Health Organization; 2020. Retrieved from https://www.who.int/publications/i/item/9789240015128.

[2] Macera CA, Powell KE. Population attributable risk: implications of physical activity dose. Med Sci Sports Exerc. 2001; 33(6 Suppl): S635–9; discussion 40–1. DOI: 10.1097/00005768-200106001-00032 11427788

[3] Macera CA, Hootman JM, Sniezek JE. Major public health benefits of physical activity. Arthritis Rheum. 2003; 49(1): 122–8. DOI: 10.1002/art.10907 12579603

[4] Macera CA. Exercise for the prevention of cardiovascular events in postmenopausal women. Clin J Sport Med. 2003; 13(2): 125–6. DOI: 10.1097/00042752-200303000-00012 12685454

[5] Hamasaki H. Daily physical activity and type 2 diabetes: a review. World J Diabetes. 2016; 7(12): 243–51. DOI: 10.4239/wjd.v7.i12.243 27350847 PMC4914832

[6] Diaz KM, Shimbo D. Physical activity and the prevention of hypertension. Curr Hypertens Rep. 2013; 15(6): 659–68. DOI: 10.1007/s11906-013-0386-8 24052212 PMC3901083

[7] Strasser B. Physical activity in obesity and metabolic syndrome. Ann N Y Acad Sci. 2013; 1281: 141–59. DOI: 10.1111/j.1749-6632.2012.06785.x 23167451 PMC3715111

[8] Blair SN, Kampert JB, Kohl, HW 3rd, Barlow CE, Macera CA, Paffenbarger, RS Jr., et al. Influences of cardiorespiratory fitness and other precursors on cardiovascular disease and all-cause mortality in men and women. JAMA. 1996; 276(3): 205–10. DOI: 10.1001/jama.1996.03540030039029 8667564

[9] Breneman CB, Polinski K, Sarzynski MA, Lavie CJ, Kokkinos PF, Ahmed A, et al. The impact of cardiorespiratory fitness levels on the risk of developing atherogenic dyslipidemia. Am J Med. 2016; 129(10): 1060–6. DOI: 10.1016/j.amjmed.2016.05.017 27288861 PMC5039056

[10] Lee IM. Physical activity and cancer prevention--data from epidemiologic studies. Med Sci Sports Exerc. 2003; 35(11): 1823–7. DOI: 10.1249/01.MSS.0000093620.27893.23 14600545

[11] Friedenreich CM, Neilson HK, Lynch BM. State of the epidemiological evidence on physical activity and cancer prevention. Eur J Cancer. 2010; 46(14): 2593–604. DOI: 10.1016/j.ejca.2010.07.028 20843488

[12] Akpan EE, Ekpenyong CE. Urbanization drift and obesity epidemic in sub-Saharan Africa: a review of the situation in Nigeria. European Journal of Sustainable Development. 2013; 2(2). DOI: 10.14207/ejsd.2013.v2n2p141

[13] Assah FK, Ekelund U, Brage S, Mbanya JC, Wareham NJ. Urbanization, physical activity, and metabolic health in sub-Saharan Africa. Diabetes Care. 2011; 34(2): 491–6. DOI: 10.2337/dc10-0990 21270205 PMC3024374

[14] Akarolo-Anthony SN, Adebamowo CA. Prevalence and correlates of leisure-time physical activity among Nigerians. BMC Public Health. 2014; 14: 529. DOI: 10.1186/1471-2458-14-529 24885080 PMC4050994

[15] Roth GA, Mensah GA, Johnson CO, Addolorato G, Ammirati E, Baddour LM, et al. Global burden of cardiovascular diseases and risk factors, 1990–2019: Update from the GBD 2019 study. J Am Coll Cardiol. 2020; 76(25): 2982–3021. DOI: 10.1016/j.jacc.2020.11.010 33309175 PMC7755038

[16] Adebamowo SN, Tekola-Ayele F, Adeyemo AA, Rotimi CN. Genomics of cardiometabolic disorders in sub-Saharan Africa. Public Health Genomics. 2017; 20(1): 9–26. DOI: 10.1159/000468535 28482349

[17] Heath GW, Parra DC, Sarmiento OL, Andersen LB, Owen N, Goenka S, et al. Evidence-based intervention in physical activity: lessons from around the world. Lancet. 2012; 380(9838): 272–81. DOI: 10.1016/S0140-6736(12)60816-2 22818939 PMC4978123

[18] Cawley J, Cisek-Gillman L, Roberts R, Cocotas C, Smith-Cook T, Bouchard, M et al. Effect of HealthCorps, a high school peer mentoring program, on youth diet and physical activity. Child Obes. 2011; 7(5): 364–71. DOI: 10.1089/chi.2011.0022

[19] Santos RG, Durksen A, Rabbanni R, Chanoine JP, Lamboo Miln A, Mayer T, et al. Effectiveness of peer-based healthy living lesson plans on anthropometric measures and physical activity in elementary school students: a cluster randomized trial. JAMA Pediatr. 2014; 168(4): 330–7. DOI: 10.1001/jamapediatrics.2013.3688 24515353

[20] Corder KL, Brown HE, Croxson CHD, Jong ST, Sharp SJ, Vignoles A, et al. A school-based, peer-led programme to increase physical activity among 13- to 14-year-old adolescents: the GoActive cluster RCT. Public Health Research. Southampton, UK; 2021. DOI: 10.3310/phr09060 33974373

[21] Buman MP, Giacobbi, PR Jr., Dzierzewski JM, Aiken Morgan A, McCrae CS, Roberts BL, et al. Peer volunteers improve long-term maintenance of physical activity with older adults: a randomized controlled trial. J Phys Act Health. 2011; r8(Suppl 2): S257–66. DOI: 10.1123/jpah.8.s2.s257 PMC3181088 21918240

[22] Castro CM, Pruitt LA, Buman MP, King AC. Physical activity program delivery by professionals versus volunteers: the TEAM randomized trial. Health Psychol. 2011; 30(3): 285–94. DOI: 10.1037/a0021980 21553972 PMC3092123

[23] Dorgo S, King GA, Bader JO, Limon JS. Comparing the effectiveness of peer mentoring and student mentoring in a 35-week fitness program for older adults. Arch Gerontol Geriatr. 2011; 52(3): 344–9. DOI: 10.1016/j.archger.2010.04.007 20537413 PMC3285567

[24] Harvard School of Public Health. Healthy eating pyramid. https://www.hsph.harvard.edu/nutritionsource/healthy-eating-pyramid/ (accessed 15 June 2022).

[25] Filmer D, Pritchett LH. Estimating wealth effects without expenditure data--or tears: an application to educational enrollments in states of India. Demography. 2001; 38(1): 115–32. DOI: 10.1353/dem.2001.0003 11227840

[26] World Health Organization. Obesity and overweight. https://www.who.int/news-room/fact-sheets/detail/obesity-and-overweight.

[27] Ainsworth BE, Haskell WL, Herrmann SD, Meckes N, Bassett, DR Jr., Tudor-Locke C, et al. Compendium of physical activities: a second update of codes and MET values. Med Sci Sports Exerc. 2011; 43(8): 1575–81. DOI: 10.1249/MSS.0b013e31821ece12 21681120

[28] Arnett DK, Blumenthal RS, Albert MA, Buroker AB, Goldberger ZD, Hahn EJ, et al. ACC/AHA guideline on the primary prevention of cardiovascular disease: A report of the American College of Cardiology/American Heart Association Task Force on clinical practice guidelines. Circulation. 2019; 140(11): e596–e646. DOI: 10.1161/CIR.0000000000000725 30879355 PMC7734661

[29] Bazzano AT, Zeldin AS, Diab IR, Garro NM, Allevato NA, Lehrer D, et al. The Healthy Lifestyle Change Program: a pilot of a community-based health promotion intervention for adults with developmental disabilities. Am J Prev Med. 2009; 37(6 Suppl 1): S201–8. DOI: 10.1016/j.amepre.2009.08.005 19896020

[30] Smith LH, Holloman C. Comparing the effects of teen mentors to adult teachers on child lifestyle behaviors and health outcomes in Appalachia. J Sch Nurs. 2013; 29(5): 386–96. DOI: 10.1177/1059840512472708 23307890 PMC5106027

[31] Spencer RA, Bower J, Kirk SF, Hancock Friesen C. Peer mentoring is associated with positive change in physical activity and aerobic fitness of grades 4, 5, and 6 students in the heart healthy kids program. Health Promot Pract. 2014; 15(6): 803–11. DOI: 10.1177/1524839914530402 24737774

[32] Tudor-Locke C, Lauzon N, Myers AM, Bell RC, Chan CB, McCargar L, et al. Effectiveness of the First Step Program delivered by professionals versus peers. J Phys Act Health. 2009; 6(4): 456–62. DOI: 10.1123/jpah.6.4.456 19842459

[33] Kraus WE, Houmard JA, Duscha BD, Knetzger KJ, Wharton MB, McCartney JS, et al. Effects of the amount and intensity of exercise on plasma lipoproteins. N Engl J Med. 2002; 347(19): 1483–92. DOI: 10.1056/NEJMoa020194 12421890

[34] O’Donovan G, Owen A, Bird SR, Kearney EM, Nevill AM, Jones DW, et al. Changes in cardiorespiratory fitness and coronary heart disease risk factors following 24 wk of moderate- or high-intensity exercise of equal energy cost. J Appl Physiol (1985). 2005; 98(5): 1619–25. DOI: 10.1152/japplphysiol.01310.2004 15640382

